# Further Reduction in Help-Seeking Behaviors Amidst Additional Barriers to Mental Health Treatment in Asian Populations: A Contemporary Review

**DOI:** 10.7759/cureus.11455

**Published:** 2020-11-12

**Authors:** Tatsuhiko Naito, Justin Chin, Tae Un Kim, Simrat Veera, Michael Jeannette, Christine M Lomiguen

**Affiliations:** 1 Psychiatry, Nuvance Health, Carmel Hamlet, USA; 2 Medical Education, Lake Erie College of Osteopathic Medicine, Erie, USA; 3 Family Medicine, LifeLong Medical Care, Richmond, USA; 4 Primary Care, Touro College of Osteopathic Medicine, New York, USA; 5 Pediatrics, Goryeb Children’s Hospital - Atlantic Health System, Morristown, USA; 6 Psychiatry, Staten Island University Hospital, New York, USA; 7 Pathology, Lake Erie College of Osteopathic Medicine, New York, USA

**Keywords:** mental health literacy, asian, help seeking behaviors, barrier, mental health treatment, culture, mental health, psychiatry and mental health, community mental health, asian american

## Abstract

Under diagnosis and treatment of mental health illnesses lead to chronic presentations and consequences. Multiple factors contribute to gaps in treatment, including the role culture plays in the development or suppression of help-seeking behaviors (HSBs). In the Asian community, conversation and recognition of mental health and its disorders are considered shameful. This review presents an analysis of literature to identify barriers to mental health treatment pronounced in Asian populations and discusses how culture influences these barriers and treatment-seeking behaviors, particularly in the context of the Asian-origin Coronavirus disease 2019 (COVID-19) global pandemic. It is the purpose of this review to discuss Asian American underutilization of mental health services and understand the factors the contribute to psychiatric care resistance in Asian communities.

## Introduction and background

Mental health incorporates an individual’s cognitive, emotional, and social well-being in the larger context of how a person perceives and interacts with their surroundings and world. Disorders of mood, thinking, and behavior are collectively grouped under mental health illness or disorders and can manifest differently for each person. Untreated mental illness can have grave consequences including pre-mature mortality, unemployment, poverty, homelessness, interpersonal conflict, co-morbid substance abuse and addiction, psychosomatic symptoms, and suicide [1]. Up to 30% of the world’s population is suffering from mental illness, yet over two-thirds of them receive no treatment. In the United States, mental illness affects 31% of the population every year, of whom 61% are untreated [2-4]. In order to reduce medical and socioeconomic burdens of mental health on society, it is paramount to investigate and address factors leading to wide gaps in treatment.

Henderson’s four barriers to mental health treatment-seeking behaviors are often cited in relation to mental health research: (1) lack of knowledge about the features and treatability of mental illness, (2) ignorance about how to access assessment and treatment, (3) prejudice against people who have mental illness, and (4) expectation of discrimination against people who have a diagnosis of mental illness [2]. One of the most often-overlooked factors, however, is the role that culture plays in the development or suppression of help-seeking behaviors (HSBs). This cannot be any more apparent in the Asian community where discussion and recognition of mental health and its disorders are considered taboo or shameful [5]. In the United States, research has shown that Asian Americans tend to underutilize mental health services relative to their severity of their symptoms [5,6]. Compared to Western populations, diasporic and American-born Asians underreport mental illnesses and are less likely to participate in mental health studies [7].

The novel and infectious betacoronavirus SARS-CoV-2, with origin most likely from an outbreak of pneumonia in Wuhan, Hubei province, China in December 2019, Coronavirus disease 2019 (COVID-19) has been declared a global pandemic by the World Health Organization (WHO) [8]. In light of these circumstances, worldwide changes to daily living to prevent the spread of infection, as recommended and imposed by federal, state, and local government guidelines, including WHO and Centers for Disease Control and Prevention (CDC) guidelines, raise Henderson’s four barriers to mental health treatment [9]. For those with mental health illnesses, it is now more challenging than ever to seek and find help. Additionally, due to the geographical origin of COVID-19 and continuous media coverage of the spread of the disease, sensationalism and discriminatory labeling has resulted in fear toward and violence against Chinese and other Asian identifying persons; creating an additional barrier for HSB: expectation of discrimination because of race or ethnicity [10-12].

HSBs and their barriers within Asian-based geographic and diaspora populations are an under-explored field within mental health research. Individual studies in various Asian subpopulations exist, however, there lacks a comprehensive review across medical literature. The purpose of this study is to review the literature in order to identify which barriers to mental health treatment are more pronounced in Asian populations and analyze the way that culture influences these barriers and treatment-seeking behaviors across the lens of Western mental health. In doing so, more culturally appropriate and targeted measures can be developed and implemented to better serve these communities.

Methods

A search was conducted of the National Library of Medicine’s Medical Literature Analysis and Retrieval System Online (MEDLINE)/PubMed, Cumulative Index to Nursing and Allied Health Literature (CINAHL), and PsycINFO databases with the objective of identifying all articles published in the English language between January 2009 and January 2019 with “help seeking behavior mental Asian,” “help seeking mental illness Asian,” and “help seeking behavior Asian”. Studies were selected to review if they were (a) published in or after 2009 in order to reflect changing attitude toward mental health in the last decade, (b) written in English, (c) primary studies, (d) full texts, (e) having either Asian or Asian American as study subjects, and (f) directly assessed either positive or negative non-demographic predicting factors of HSB (i.e., literature that merely mention predicting factors in introduction but do not directly assess them were excluded). Studies solely analyzing college students seeking help from university-run health centers or those solely focused on the pediatric population were excluded for their subjects not being representative of the general population. Study protocols or pilot work without data or results were also excluded. All pertinent literature was retrieved, analyzed, and thoroughly searched in order to identify any potential additional manuscripts that could be referenced.

The comprehensive search revealed a total of 804 manuscripts, of which duplicates or articles not of the English language were also excluded. This yielded a total of 18 manuscripts that were completely assessed and incorporated into this review. Each article was coded based on Henderson’s four barriers to mental HSBs. Of note, “Asian” is defined in this review as those of Eastern or South Asian descent, regardless of their nationality, immigration status, or current residing country.

## Review

Results

Qualitative data for the comprehensive review of the 18 articles in regard to the HSB by country and type can be found in Table 1.

**Table 1:** Review of literature regarding Asian mental health help-seeking behaviors with subjective Henderson classification Henderson Classification: (1) lack of knowledge about the features and treatability of mental illness, (2) ignorance about how to access assessment and treatment, (3) prejudice against people who have mental illness, (4) expectation of discrimination against people who have a diagnosis of mental illness, and (5) other types of barrier.

Author	Ethnicity of participants	Study location	Psychiatric condition	Special consideration	Henderson Classification
Luitel et al. [1]	Nepali	Nepal (Chitwan)	Depression / Alcohol use disorder	N/A	(1), (2), (3), (5)
Yu et al. [4]	Chinese	China (Liuyang)	Not specified	N/A	(1), (2), (3), (4), (5)
Found [13]	Chinese	Macao	Not specified	Age over 60	(3)
Kim-Mozeleski et al. [14]	Vietnamese / Vietnamese American	USA (San Francisco/ Washington DC)	Depression	N/A	(3), (5)
Nguyen [15]	Asian American (unspecified)	USA (California)	Not specified	Age over 50	(5)
Qui et al. [16]	Chinese	China (Guangyuan)	Depression	Only women	(1), (3), (5)
Suka et al. [17]	Japanese	Japan	Depression	N/A	(1), (5)
Ta Park et al. [18]	Chinese American	USA (Northern California)	Postpartum depression	Married women of childbearing age	(1), (3), (5)
Ta Park et al. [19]	Vietnamese American	USA (Northern California)	Postpartum depression	Majority of subjects were immigrants	(1), (2), (3)
Chen et al. [20]	Taiwanese	Taiwan	Panic disorder	N/A	(3)
Goyal et al. [21]	Indian	USA (north California)	Postpartum depression	Only others between 29-40	(3), (5)
Liu et al. [22]	Chinese	Hong Kong	Insomnia	N/A	(1), (2)
Maekawa [23]	Japanese	Japan	“Distress”	Working males	(3), (4), (5)
Sorkin et al. [24]	Asian American (unspecified)	USA (California)	Not specified	N/A	(3), (5)
Stariton et al. [25]	Filipino	Norway	Not specified	Immigrants	(1), (2), (3)
Suka et al. [26]	Japanese	Japan	Suicidality	N/A	(1), (2), (3)
Wales et al. [27]	South Asian (Asian/British Asian Indian, Pakistani or Bangladeshi)	United Kingdom (Leicester)	Eating disorder	N/A	(1), (3), (5)
Wu et al. [28]	Taiwanese	Taiwan	Anxiety/depression	N/A	(1), (3)

Ethnicity of subjects in these studies included Chinese (5), Japanese (3), Vietnamese (2), Taiwanese (2), Nepali (1), Indian (1), Filipino (1), and broad categories of South Asian (1) and Asian American (2). Location of these studies is spread out geographically from the United States to countries in Asia and Europe. All of the studies conducted in the United States were completed in the state of California. Six out of 18 studies (33%) focused on participants’ attitude or HSB toward nonspecific mental illness. The remaining 12 (67%) studies covered HSB toward: major depressive disorders, postpartum depression, alcohol use disorder, panic disorder, suicidality, insomnia, and eating disorder. Eight of the 18 studies (44%) selected participants with specific demographics such as age over 50, immigrated to country of study, or married women of childbearing age.

HSBs are defined as planned, problem-focused interpersonal interactions involving a healthcare professional and a patient [29]. Broadly dependent on the attitudes (beliefs and willingness) towards seeking help, HSB also includes the intention, results, and subsequent performance of the behavior [30]. In patients with suspected mental illness, decreased HSB has been associated with treatment avoidance, noncompliance, and failure. The etiology of decreased HSB has been broadly classified into Henderson’s four barriers to mental health treatment, by which this review categorizes the aforementioned articles [2]. Of the four barriers, perceived prejudice/stigma for those with mental health illnesses was the most prevalent, followed by a lack of knowledge in mental health and other factors. Unsurprisingly, many of the studies detail barriers that are outside of Henderson’s classification, which underscores the complexity and multifaceted nature of mental health in Asian communities.

Lack of knowledge about the features and treatability of mental illness

Mental health literacy (MHL) includes the knowledge and beliefs surrounding the recognition, management, and prevention of mental health illnesses [4,31]. Of all the components of MHL, the ability to recognize symptoms is considered to be the most important, as it transitions patients from a pre-contemplative to a contemplative phase when seeking help [4]. MHL was initially built from, and still remains largely, a Western perspective of mental illness and hence necessitates a culturally informed framework tailored to Asian communities [14]. Due to the varied manifestation of mental illnesses, ranging from psychological to somatic, recognition has been shown to be crucial in seeking professional help, while those lacking this ability are more commonly associated with delays in seeking help [4,26]. Another predictive factor for positive mental HSB is whether patients have insight into the treatability of mental illnesses as recognition alone may not always equate to motivation to seek treatment [32].

Of 18 studies incorporated into this review, 10 (56%) of the studies showed either knowledge about the features or treatability of mental illness being a positive predictor of mental health HSB or lack thereof being a barrier (i.e., negative predictor) to HSB. Five studies did not assess for this particular factor while 1 indicated that there was not a significant association. In the majority of studies, those with lower MHL were less likely to seek help for mental illness, with Asian populations having disproportionately lower MHL compared to other racial groups. Participants commonly exhibited a lack of general understanding of their respective mental illnesses or did not deem their symptoms severe enough to seek treatment [19,22,25,28]. Although not all studies elaborated on the particular motivations of participants, contextualized analysis demonstrated the unique ways that culture can undermine MHL in Asian populations. Concepts of mental illness simply did not exist in their culture hence compromising their ability to recognize or attribute their symptoms to their mental state. For example, mental health issues are collectively grouped under “Xinxiang bing” in Chinese or described symptomatically as being sad or tired. Particularly prevalent in rural locales, many participants believe mental health is a Western concept and in turn, failed to utilize available resources for treatment [16].

Ignorance about how to access assessment and treatment

Despite having insight into their condition, a lack of knowledge regarding treatment options and how to access them can be a large impediment to developing positive HSBs [33]. Lack of awareness or knowledge about existing services has been associated with underutilization of mental health services in previous studies, especially in Asian populations [34]. Of 18 studies incorporated to this review, six listed the lack of ideas about resources as strong or one of the most commonly expressed deterrents to seek help from mental health professionals among their participants. In particular, during the COVID-19 pandemic, it is important for all individuals to be aware of telehealth options for mental health treatment, as digital technology has the ability to alleviate isolation and act as a bridge in times of social distance [35,36]. One of the most commonly mentioned barriers, outside of Henderson’s four, is the general lack of access and targeted services for the Asian population. Noted in 10 of the 18 studies, this obstacle manifested in various ways ranging from socioeconomic restrictions such as lack of financial or insurance coverage to structural inequalities such as a lack of mental health infrastructure in their area [1,16,25,26]. Cultural competence was a critical factor as participants expressed concerns about the lack of mental health professionals who are both culturally and linguistically capable [14,18,19,21,25,37].

Extending beyond physician knowledge and competency, language presents another complicated layer in parsing out the role the culture plays in the mental health HSBs. For example, in a study of Filipino immigrants and Norwegian physicians, in which both groups could proficiently communicate in English, Filipino participants still felt that language barriers existed when discussing the complex nature of mental illness [25]. While able to describe such issues in Tagalog, many of the subtleties and nuances were lost in translation, leading to inadequate or incorrect assessments of the participants’ mental health status. Such findings emphasize the importance of mental health professionals being able to speak the same language as patients and having the cultural competency to address any preconceived societal thoughts about mental illness, which ultimately affects a patient’s motivation to seek and stay with treatment. Notwithstanding these barriers, it is important to also reflect on the cognitive effects of mental illness on the perception of available resources. In another study, HSB was compromised by depressive symptoms despite abundance of appropriate resources and awareness in Japan [23]. Further investigation into the role that culture can play into these barriers is warranted.

Prejudice against people who have mental illness

Prejudice or stigma against people who have mental illness is one of the most well-researched areas of mental health studies, with significant documentation establishing its negative effects on HSB [2,4,16,28,34]. While stigma surrounding mental health is hardly unique to a singular ethnic or racial group, Asian and Asian American communities have been found to have higher than average prejudices toward those with mental illness [34]. Of the 18 studies, 15 cited prejudice or stigma as the primary reason in avoiding treatment, which was, by far, the most prevalent barrier among Henderson’s four barriers. On further elaboration, participants mentioned the fear of being perceived as crazy, saving “face”, and fear of bringing shame to their family as common themes [1,3,13,14,16,18,21,25,26]. In seven of the 18 studies, mental illness was attributed to personal incompetence or weakness, in which those suffering did not have the mental fortitude or resilience to cope with their own problems [4, 25]. In such cases, there is an element of double stigmatization as the individual would not only be negatively labelled by society, but also seen as inadequate in resolving their own problems if treatment or help is sought [25]. As seen in the study populations, this notion is pervasive across multiple Asian cultures including Nepali, Vietnamese, Chinese, Indian, and Filipino, each of which have unique sociohistorical and geopolitical customs that shape their understanding of mental health.

Study participants highlighted an aversion to seeing or being referred to mental health professionals, especially in smaller communities where people are more likely to know each other. Somatization, in which psychological distress is manifested in unexplained physical presentations, is common in the Asian population as there is a tendency to focus on the physical symptoms. Somatic complaints are often perceived as more important due to the emphasis and amplification of physiological changes and their central role in daily lives, while also being free of the stigma associated with mental complaints [4]. For example, in Indian communities, feelings of weakness or fatigue (kamzori) are more accepted than that of depression [34]. Coupled with the overwhelming preference of seeing their primary care physician or a traditional healer over one specializing in mental health, misdiagnosis and undertreatment for mental illness are common for Asian patients. For many of the participants, it was more culturally accepted to admit physical symptoms rather than psychological symptoms, which ultimately lead to the preference of seeing primary care doctors over mental health professionals such as psychiatrists [4,14,26,34].

Ultimately, this emphasizes the contributions from not only psychiatrists but also from primary care doctors in educating and eradicating stigma toward mental illness among Asian communities. Several studies in this review mentioned participants' inclination to discuss their psychological symptoms with family members over mental health professionals [14,21,25]. This is explained by the tendency of individuals with roots in Asian cultures typically preferring to keep information about family problems kept in confidence within this kinship domain due to stigmatization [34]. Positive perception of family and friends regarding help-seeking was significantly associated with the help-seeking intention from professionals [26]. This presents family/friends as another possible area for intervention to enhance mental health HSB in the Asian community.

Expectation of discrimination against people diagnosed with mental illness

Anticipated discrimination is closely related to stigma and fear of public perception, which is a defining feature for decreased HSBs in the Asian community. The distinction between experienced and anticipated discrimination is subtle such that they are often intertwined, resulting in lowered self-esteem and a cyclical pattern between fear and further development of mental illness [38]. 2 out of 18 studies cited anticipated discrimination as a deterrent to HSB, especially in relation to potential effects on their career or social standing in the community [4, 23].

Psychoeducation of the population, in both clinical and non-clinical settings, is paramount in improving MHL and eradicating stigma in the Asian community. Specifically, educators must take elements such as cultural beliefs about the etiology of mental disorders, including those associated with stigma (i.e. attributing personal weakness as a cause of mental illness), into the account, as it has been established as important predictors of HSB [13]. In addition, community outreach through medical and non-medical channels are needed to take a role in educating the community about the availability of formal resources. Several studies note that despite such importance of community based, culturally oriented education programs or campaigns, these practices are still scarce, hence need to be addressed by policymakers and supportive legislation [14, 26]. As evinced in this review, cultural competency training and recruitment of physicians versed in Asian culture is severely lacking in academic and medical settings. With a high tendency among Asian populations to somaticize mental illness and resulting preference to see family doctors first, this applies not only to psychiatrists but also to primary care doctors as well. Specifically, these health professionals must be able to make culturally appropriate adjustments in the counseling sessions and treatment plans [5,6,13,18,19].

There are several limitations to this review. Each subpopulation within the larger Asian classification has different cultures, values, and histories, such that it is not appropriate to overgeneralize or homogenize findings for the entire group. All of the studies utilized different scales, metrics, and formats (qualitative vs quantitative) to assess the HSBs. Various studies also examined narrow niche groups as subjects, such as only assessing women of childbearing age. As a whole, this review serves as a reminder and clarion call for increased research and attention to mental health in Asian communities, especially when many cultural customs consider keeping problems “in-house” as culturally virtuous [26].

Expectation of discrimination against people because of race or ethnicity

**Figure 1:**
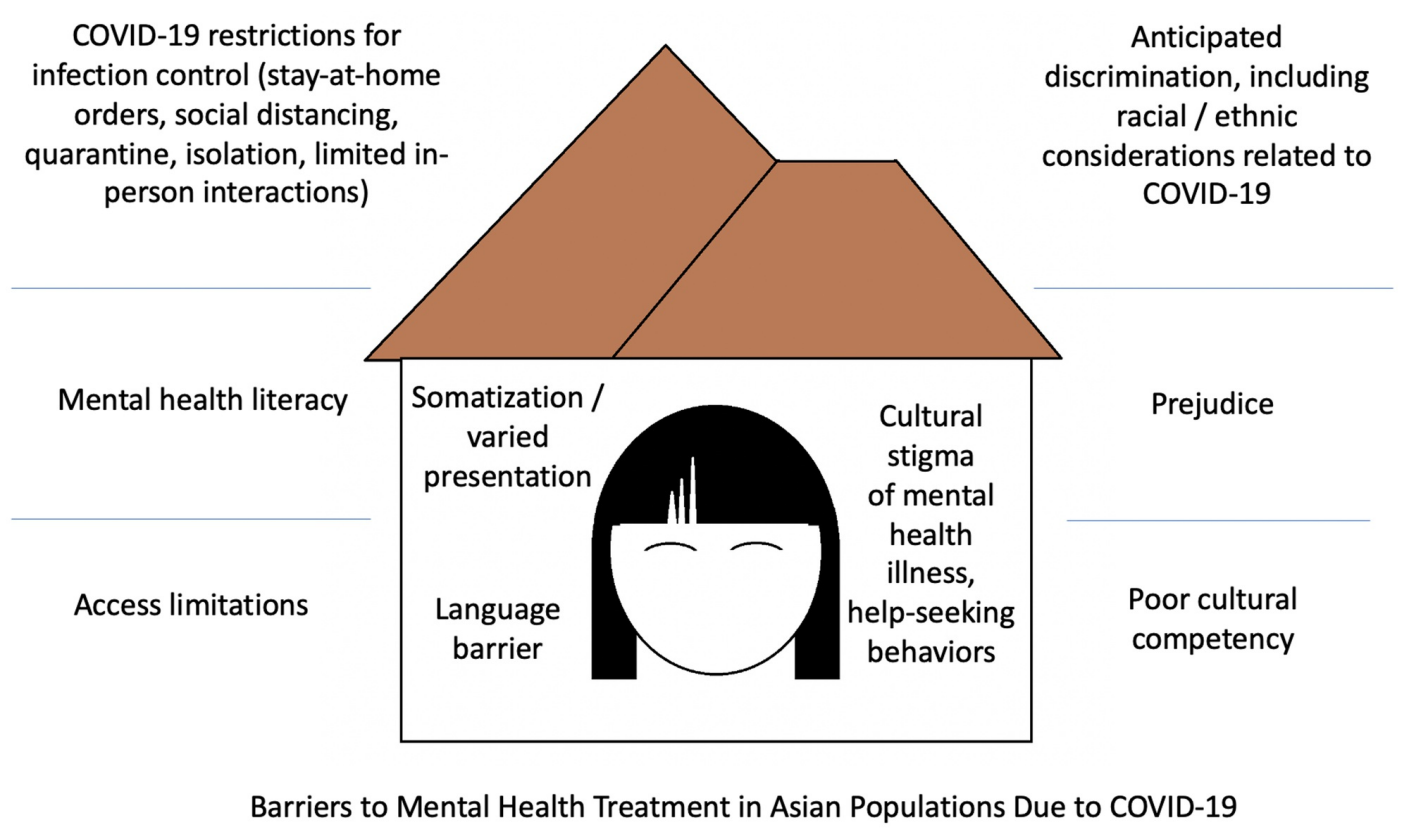
Visual representation of the potential barriers to mental health treatment in Asian populations due to the COVID-19 pandemic Original graphic by Christine Lomiguen.

Due to the likely Chinese origin of COVID-19 and continuous media coverage of the spread of the disease, sensationalism and discriminatory labeling has resulted in fear toward and violence against Chinese and other Asian persons, creating this additional barrier for HSB: expectation of discrimination because of race or ethnicity [10-12]. A recent study determined various forms of social exclusion and discrimination, including but not limited to, verbal abuse, violent attacks, laid off from employment without proper cause, occurred toward Chinese persons living both in mainland China and overseas during the COVID-19 pandemic [39]. Social stigma, exacerbated by high-ranking politicians grounded on general public fear of disease unknown, reduces the likelihood of HSBs not only for possible infectious disease, like COVID-19, but also can be extended for mental health disease [40]. Additional research into the mental health and HSB ramifications are required during and post-COVID-19 pandemic (Figure 1).

## Conclusions

Barriers to mental health HSBs in Asian communities are complicated topics to parse as each barrier is not mutually exclusive but rather intertwined to each other. Focusing on a singular barrier such as lack of resources, is often not enough as Asian communities often have multiple barriers that contribute to each other to manifest in the presenting patient. Somatization is a common cultural theme seen in the Asian community, which can stem from a lack of knowledge in mental illness symptoms, stigma, or both. Greater research on the interaction of HSBs with culture is needed as this can have larger implications for developing better public health programs and improving quality of care by enabling more culturally competent training for healthcare workers, even more so in the context of the Asian-origin COVID-19 global pandemic.
